# P-195. Clinical *Clostridioides difficile* Diagnostic Test Types and Results are Associated with the Likelihood of Recovering *C. difficile* in Stool Cultures

**DOI:** 10.1093/ofid/ofae631.399

**Published:** 2025-01-29

**Authors:** Andrew M Skinner, Alice Guh, Laurica A Petrella, Susan P Sambol, Stuart Johnson, Christopher A Czaja, Helen Johnston, Elizabeth Basiliere, Robin Dhonau, Lauren C Korhonen, Ashley Paulick, Michelle Adamczyk, L Clifford McDonald, Dale N Gerding

**Affiliations:** University of Utah, Salt Lake City, Utah; Centers for Disease Control and Prevention, Atlanta, Georgia; Edward Hines Jr VA Hospital, Hines, Illinois; Edward Hines Jr. VA, Hospital, Hines, Illinois; Hines VA Hospital and Loyola University Medical Center, Hines, Illinois; Colorado Department of Public Health and Environment, Denver, Colorado; Colorado Department of Public Health, Denver, Colorado; Colorado Department of Public Health and Environment, Denver, Colorado; Georgia Emerging Infections Program, Decatur, GA; Emory University School of Medicine, Atlanta, GA; Atlanta Veterans Affairs Medical Center, Decatur, GA, Brookhaven, Georgia; CDC, Atlanta, Georgia; Centers for Disease Control and Prevention, Atlanta, Georgia; CDC, Atlanta, Georgia; CDC, Atlanta, Georgia; Edward Hines, Jr. Veterans Affairs Hospital, Hines, Illinois

## Abstract

**Background:**

Active *Clostridioides difficile* infection (CDI) surveillance is conducted by the U.S. CDC’s Emerging Infections Program (EIP) and includes collection of a convenience sample of stool specimens from *C. difficile* positive cases. Accurate molecular epidemiology relies on recovering *C. difficile* from clinical specimens. We sought to determine whether CDI diagnostic testing approach was associated with the likelihood of culturing *C. difficile* from stool.

**Table 1.**
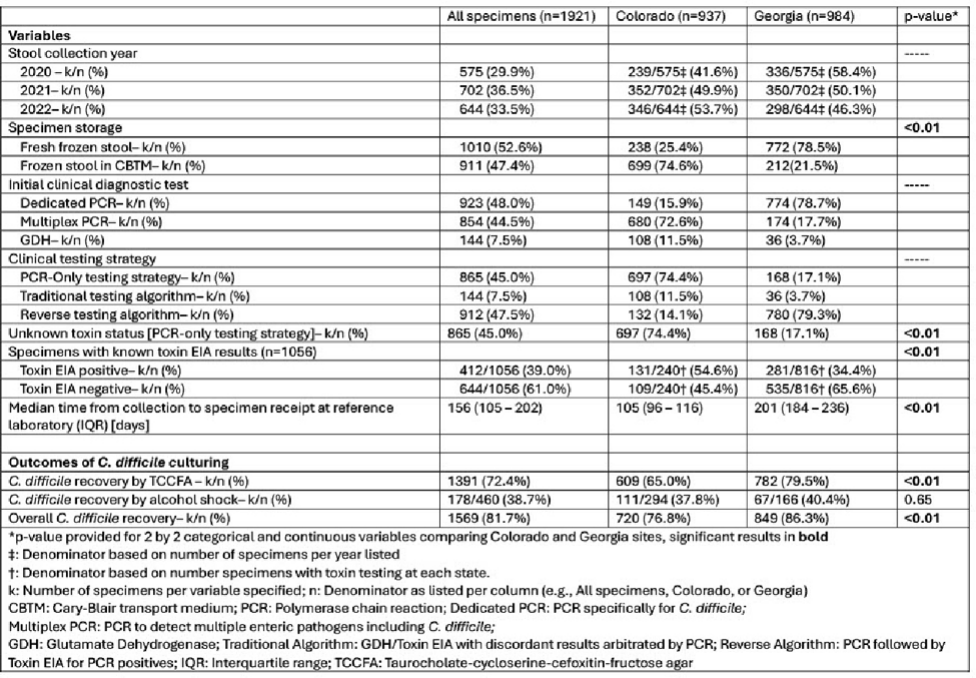
Characteristics of stool specimens collected from Clostridioides difficile positive stool specimens identified through Emerging Infections Program surveillance in Colorado and Georgia, January 2020 through December 2022

**Methods:**

We performed a cross-sectional study of the recovery of *C. difficile* from *C. difficile* positive stools collected from 1/2020 – 12/2022 from the Colorado and Georgia EIP sites. Stools were collected and stored per site protocols (i.e. Cary-Blair transport medium [CBTM] or fresh stool), frozen, and cultured at a reference laboratory. Multivariable logistic regression models were constructed to determine the adjusted odds ratio (aOR) for key variables in relationship to *C. difficile* recovery. A separate semi-quantitative experiment evaluating the impact of CBTM on recovery was conducted by placing stools in which *C. difficile* had been recovered into CBTM vs. phosphate-buffered saline (PBS).

**Table 2.**
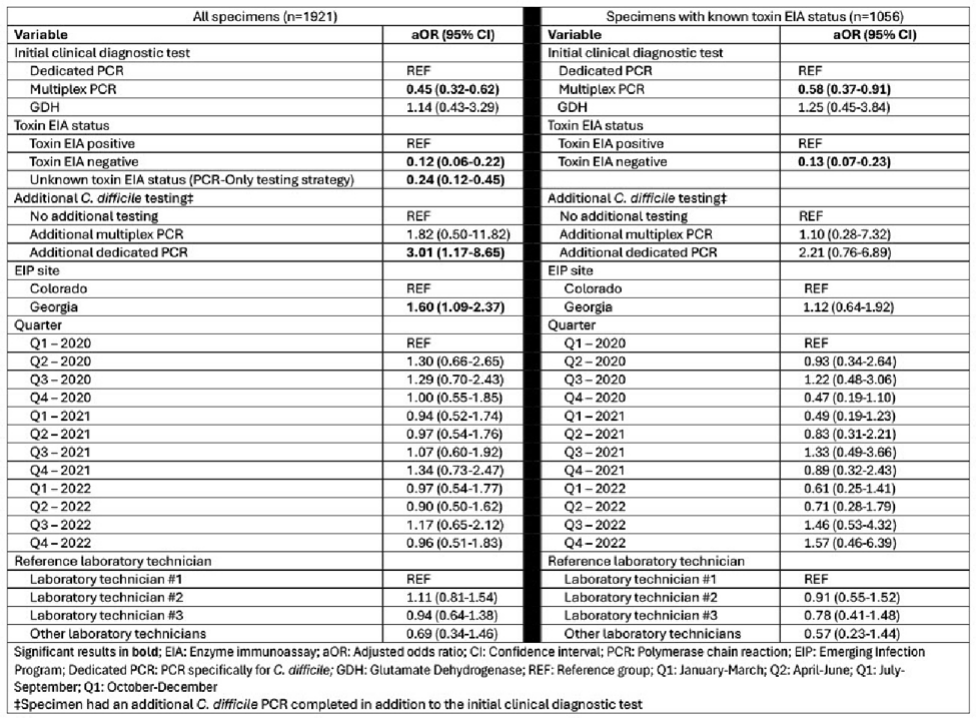
Multivariable logistic regression models for Clostridioides difficile recovery from C. difficile positive stool specimens identified through Emerging Infections Program surveillance in Colorado and Georgia, January 2020 through December 2022

**Results:**

*C. difficile* was cultured from 1569 of 1921 stools (81.7%, Table 1). Semi-quantitative experiments revealed that CBTM had no impact on *C. difficile* recovery. Recovery of C. *difficile* from stool cultures was less likely among cases in which *C. difficile* was detected: 1) by multiplex PCR (aOR 0.45, 95% confidence interval [CI]: 0.32-0.62); 2) with a negative toxin enzyme immunoassay (EIA) (aOR 0.12, 95% CI: 0.06-0.22); and 3) by a PCR-only testing strategy (aOR 0.24, 95% CI: 0.12-0.45). Among 1056 cases with known toxin EIA test results*, C. difficile* recovery was less likely when *C. difficile* detection was associated with a multiplex PCR and in those with a negative toxin EIA (Table 2).

**Conclusion:**

The ability to culture *C. difficile* from stool specimens was reduced when *C. difficile* was detected with a multiplex PCR, a PCR-only testing strategy, or with negative toxin EIA results. These findings can be used to maximize the yield of surveillance stool submission and culture protocols, thereby enhancing the ability to generate robust molecular epidemiology data to inform prevention and control efforts.

**Disclosures:**

**Andrew M. Skinner, MD**, BioK plus: Advisor/Consultant|Ferring Pharmaceuticals: Advisor/Consultant|Recursion pharmaceutical: Advisor/Consultant **Stuart Johnson, M.D.**, Acurx Pharmaceuticals: Advisor/Consultant|Bio-K Plus International: Advisor/Consultant|Ferring Phamraceuticals: Advisor/Consultant **Dale N. Gerding, MD**, AstraZeneca: Advisor/Consultant|Destiny Pharma: Advisor/Consultant|Destiny Pharma: Licensed IP to Destiny|Sebela: Advisor/Consultant|Sebela: Licensed IP

